# Patulous Eustachian Tube and Otitis Media With Antineutrophil Cytoplasmic Antibody-Associated Vasculitis (OMAAV)

**DOI:** 10.7759/cureus.53677

**Published:** 2024-02-05

**Authors:** Iori Kusaka, Ryoukichi Ikeda, Aya Katsura, Kiyoto Shiga

**Affiliations:** 1 Department of Otolaryngology, Head and Neck Surgery, Iwate Medical University School of Medicine, Yahaba, JPN

**Keywords:** acute otitis media, antineutrophil cytoplasmic antibody (anca) associated vasculitis (aav), chronic otitis media, patulous eustachian tube, eustachian tube function

## Abstract

Antineutrophil cytoplasmic antibody (ANCA)-associated vasculitis is a systemic necrotizing vasculitis that affects small to medium-sized vessels. We describe two cases of patulous Eustachian tube (PET) in patients with otitis media with ANCA-associated vasculitis (OMAAV). The two cases presented in this paper had previously been diagnosed with Eustachian tube (ET) stenosis, and both presented with bilateral aural fullness, with one also experiencing postnasal drip and hearing loss. Both patients experienced positive myeloperoxidase (MPO)-ANCA and negative proteinase 3 (PR3)-ANCA, and treatment for ANCA-associated vasculitis (AAV) resulted in a diagnosis of PET. The patients were treated with transnasal self-installation of physiological saline into the pharyngeal orifice of the ET. This paper highlights the importance of considering PET in the differential diagnosis of OMAAV patients presenting with aural fullness.

## Introduction

Antineutrophil cytoplasmic antibody (ANCA)-associated vasculitis is a systemic necrotizing vasculitis that affects small to medium-sized vessels. ANCA-associated vasculitis is classified into three categories, granulomatosis with polyangiitis (GPA), microscopic polyangiitis (MPA), and eosinophilic granulomatosis with polyangiitis (EGPA) [1, 2]. Recently, the Japan Otological Society (JOS) proposed “otitis media with ANCA-associated vasculitis (OMAAV)” which includes otitis media caused by GPA, MPA, and EGPA, as well as otitis media that does not fulfill the ordinary diagnostic criteria for systemic ANCA-associated vasculitis [3, 4].

OMAAV is more common in women (71%) and middle age (median age 67 years). The occurrence of myeloperoxidase (MPO)-ANCA positivity is highest at 56%, followed by proteinase 3 (PR3)-ANCA positivity at 23%, but the incidence of negativity for both variants of ANCAs is 17%. The complications include facial nerve palsy and hypertrophic pachymeningitis [4]. OMAAV can be classified as the otitis media with effusion (OME) type, and the otitis media with granuloma (OMG) type based on findings in the tympanic membrane [5]. Sahyouni et al. reported a series of patients who were eventually diagnosed with GPA based on their otologic complaints. All patients presented with ET stenosis, OME, or both [6]. In a nationwide survey conducted in Japan, OME and OMG were observed in 49% and 44% of OMAAV patients, respectively [4]. There were no appreciable between-group variations in clinical symptoms/signs, ANCA status, sequelae, or prognosis. However, there have been no reports of patulous Eustachian tube (PET) with OMAAV. Patients with PET complain of annoying aural symptoms including voice and breathing autophony as well as aural fullness caused by prolonged ET patency, and frequently experience a decrease in quality of life [7]. PET is often mistaken for ET stenosis (dysfunction) because it presents with aural fullness [8-10]. Herein, we present two unique cases of PET with OMAAV.

## Case presentation

Case 1

**Figure 1:**
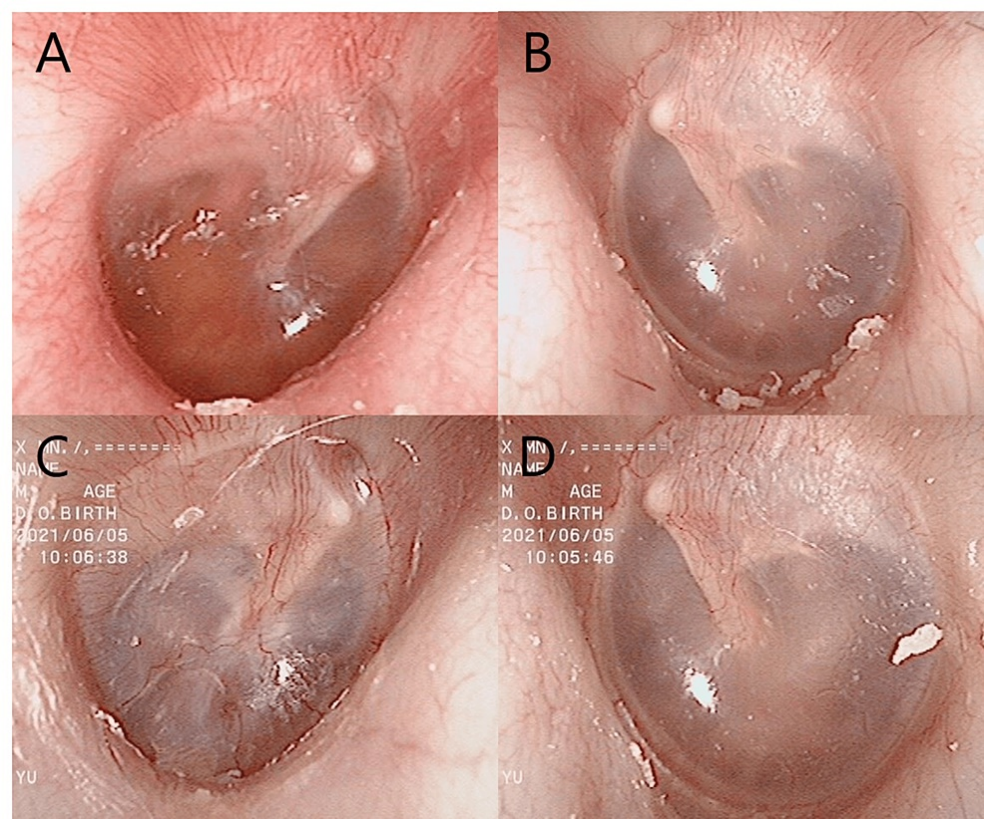
Tympanic membrane findings at the first visit (A: right ear, B: left ear) and after treatment for OMAAV (C: right ear, D: left ear) in case 1. Middle ear effusion was observed (A).

A male patient, aged 58, presented to our hospital with bilateral aural fullness and was referred to our department for evaluation of his aural symptoms. His medical history included hypertension, hyperuricemia, hyperlipidemia, femur fracture, and hallux valgus. He was previously diagnosed with ET stenosis but discontinued his hospital visits. Two years later, he returned to our department with intermittent bilateral aural fullness, which was more prominent on the right side. The right tympanic membrane showed middle ear effusion (Figure 1A), and the left tympanic membrane seemed to be without effusion (Figure 1B). Tympanostomies were performed on his right ear several times, but unfortunately, his symptoms did not improve, and he also experienced purulent nasal discharge and headache. A blood sample was collected to investigate the possibility of intractable otitis media. The blood test revealed 1.0% eosinophils, a positive result for MPO-ANCA (113 IU/mL; normal range<3.5 IU/mL), and negative for PR3-ANCA (normal range<3.5 IU/mL). As a result, he was referred to the Department of Collagen Disease in our hospital and was diagnosed with ANCA-associated vasculitis. The patient received IV cyclophosphamide (1200 mg every four weeks) and prednisone (initial dose 80 mg/day) for two months, resulting in a 12 kg weight loss. Three months after starting treatment, the patient reported left voice autophony and aural fullness, which was relieved in the supine position, along with the respiratory fluctuation of the bilateral tympanic membrane (Figures 1C-1D).

**Figure 2:**
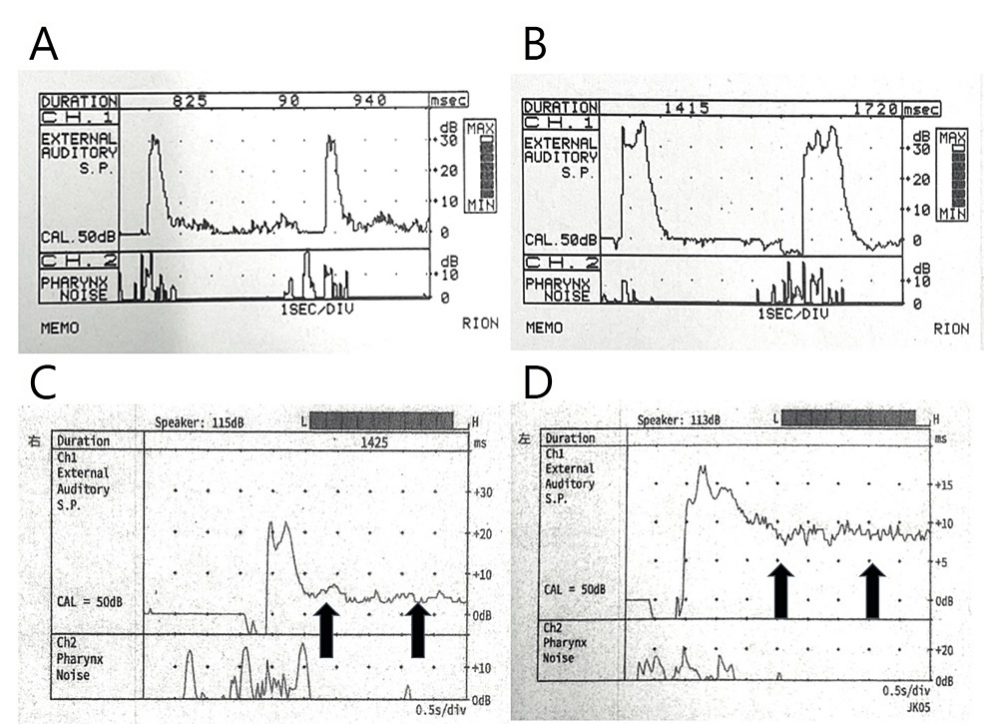
Sonotubometry results in case 1 (A: right ear, B: left ear) and case 2 (C: right ear, D: left ear). Sonotubometry analyzes sound transmission from the nasopharynx (lower rows) to the EAC (upper rows) via the ET. In the sonotubometry mode, the input sound pressure level is automatically created (speaker level). A 7 kHz octave band noise at the nostril makes up the acoustic signal, and this noise causes the EAC to produce an output with a preset 50 dB SPL. The ET opening during swallowing was associated with a prolonged increase in EAC pressure and a decrease in probe tone SPL, both of which were indicative of positive PET results (plateau-shaped waveform: black arrow).

Sonotubometry showed normal findings (Figures 2A-2B).

A diagnosis of left PET was made based on the Otological Society of Japan (JOS) Diagnostic Criteria for the patulous Eustachian tube [11]. The patient was treated with transnasal self-administration of physiological saline in the pharyngeal orifice of the ET therapy for six months [12,13]. His symptoms did not improve, so the patient was referred to another hospital for silicone plug surgery.

Case 2

**Figure 3:**
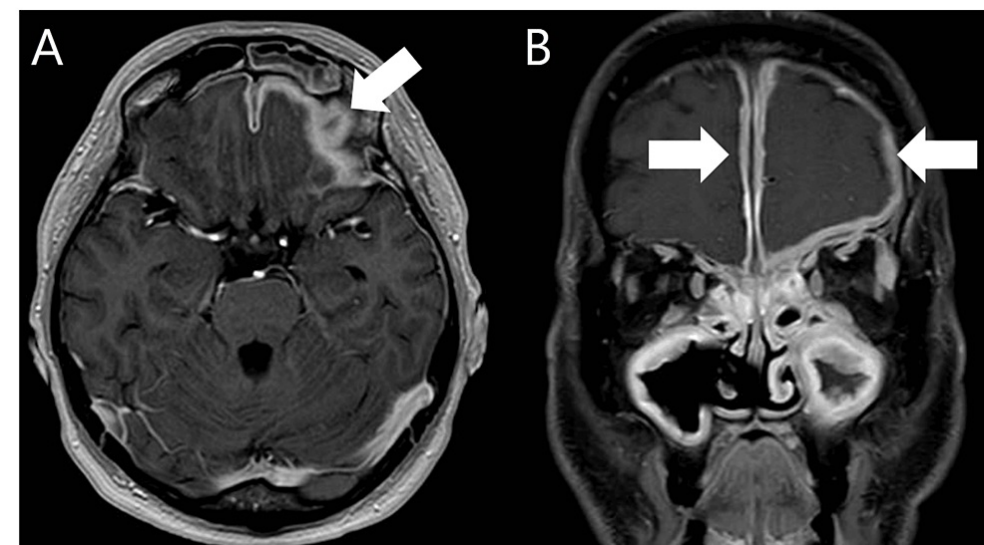
T1-weighted MRI images (left: axial, right coronal). T1-weighted MRI images showed that the dura thickened and contrasted with gadolinium (Gd) (white arrow).

A male patient, aged 50, noticed bilateral aural fullness, and one year later, visited an otolaryngologist at another hospital because his aural fullness had worsened, and he was experiencing hearing loss (Right ear: 50dB, Left ear: 45dB) and postnasal drip. His aural symptoms were temporarily improved by sniffing. Bilateral tympanostomies were performed, which provided temporary relief of his aural fullness. However, his symptoms returned later. A year after that, he began to experience foggy vision in his left eye. Computed tomography (CT) revealed bone non-thinning, loss, and destruction in both skull bases, the right medial orbital wall, and the left orbital floor. Contrast-enhanced magnetic resonance imaging (MRI) suggested hypertrophic pachymeningitis (Figure 3), and blood tests revealed a positive result for MPO-ANCA (91 IU/mL) and a negative result for PR3-ANCA.

**Figure 4:**
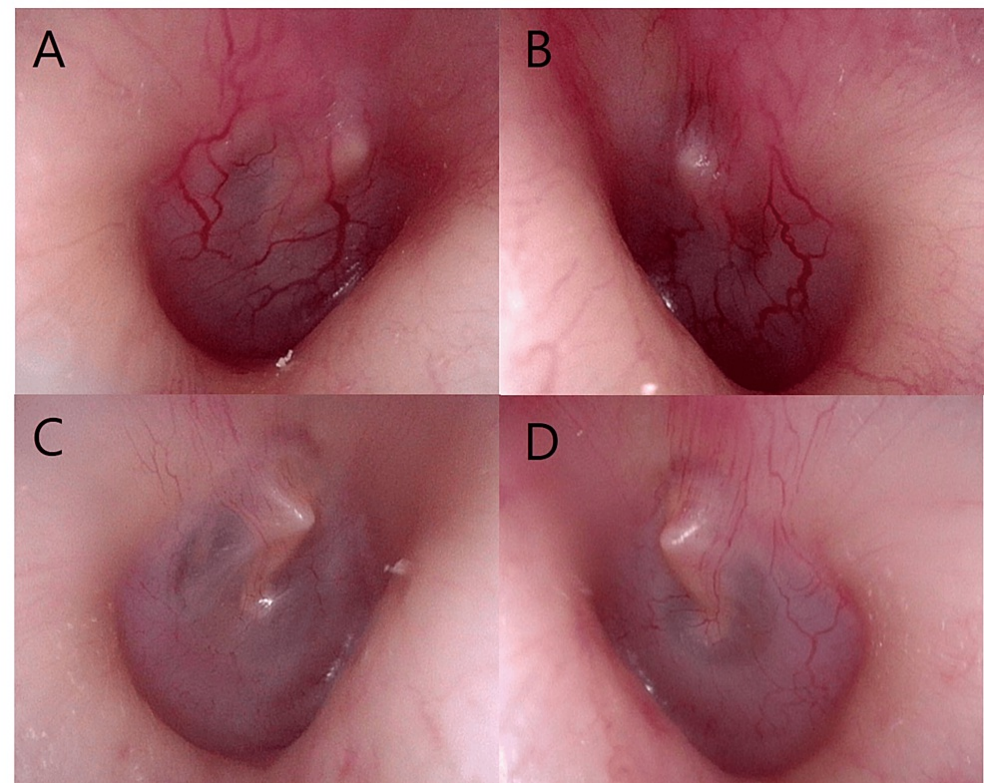
Tympanic membrane findings at the first visit (A: right ear, B: left ear) and after treatment for OMAAV (C: right ear, D: left ear) in case 2. Vasodilation of the tympanic membrane and external auditory canal improved with treatment.

Consequently, he underwent endoscopic nasal and sinus surgery, and biopsies of the bilateral maxillary sinuses, ethmoid sinus, and maxillary sinus mucosa were performed. The histological studies showed no evidence of vasculitis. Based on his symptoms and examination results, ANCA-associated vasculitis was suspected, and the patient was referred to our department and the Department of Collagen Disease for further examination and treatment (Figures 4A-4B). The patient received IV cyclophosphamide (1200 mg every two weeks) and prednisone (initial dose 80 mg/day) for three months. After starting treatment for ANCA-associated vasculitis, the patient reported bilateral voice autophony and aural fullness, and respiratory fluctuation of the right tympanic membrane was observed (Figures 4C-4D).

His body weight decreased by 10 kg. Sonotubometry revealed bilateral open plateau type (Figures 2C-2D), confirming the diagnosis of PET. The patient was treated with transnasal self-installation of physiological saline to the pharyngeal orifice of the ET. Aural symptoms of PET have been well controlled by this treatment.　

## Discussion

We have presented two unique cases of OMAAV patients with PET. OMAAV can be classified into two types, OME or OMG, based on the observation of the tympanic membrane, which displays tympanic membrane enlargement due to granulation. Retained fluid can be observed through the tympanic membrane in cases where a tympanostomy is performed or when OME type is present. Inflammatory manifestations such as tympanic membrane thickness, edema, and redness were apparent in the OMG type. Myringotomy revealed granulation tissue in the middle ear. In some patients, the tympanic membrane may not be visualized due to swollen external auditory canal, while in others, dilated capillaries from the external auditory canal to the posterior portion are observed [4, 5]. Tympanic membrane thickness, vasodilation, and edema of the posterior portion are typical characteristics of the tympanic membrane in OMAAV patients [14]. At the initial examination, Case 1 displayed OME in the right ear, while Case 2 showed thickening and vasodilation of the tympanic membrane. At the time of PET diagnosis, Case 1 had a normal tympanic membrane, while Case 2 had mild thickening and vasodilation. Particularly in cases such as Case 2, where findings such as vasodilation of the tympanic membrane were present, it may be difficult to consider the differential disease of PET, and careful attention should be paid to these cases. In addition, it is highly likely that the Case 2 patient had developed PET before OMAAV treatment since sniffing had improved the aural fullness before treatment. The diagnosis of PET is established based on aural symptoms, the movement of the tympanic membrane upon respiration, and ET function tests [7, 11, 15, 16]. PET symptoms should be eliminated or alleviated by obstructing the open ET during symptom manifestation. Our case may not be rare, but rather, it is more likely that PET is not being properly diagnosed [17]. Further study is needed to determine what proportion of PET cases are present in OMAAV. Aural fullness is usually observed not only in ET stenosis but PET [9]. Perhaps it is more likely that the diagnosis of PET is not properly made rather than that our case is rare.

There are a number of possible mechanisms through which OMAAV can lead to PET. Firstly, body weight loss due to ANCA-associated vasculitis causes PET. Patients with ANCA-associated vasculitis typically exhibit systemic signs and symptoms including fever and weight loss [1]. Our patients, Cases 1 and 2, lost 12 kg and 10 kg, respectively. Bodyweight loss, caused by the volume loss of tissues including Ostmann's fatty tissue and glandular tissue in the cartilaginous section, is a common risk factor associated with PET [7, 9]. These findings are consistent with the Ostmann fat pad's atrophic changes in PET patients. Ostmann's fat pad has a maximum thickness of around 20 mm dorsolateral of the pharyngeal orifice and gradually gets thinner as it gets closer to the orifice [18]. It was demonstrated that in PET patients using 3 tesla MRI, it was much smaller [19]. Secondly, another potential cause is the inflammation of the ET mucosa due to ANCA-associated vasculitis, which may lead to ET patency due to atrophy of the ET mucosa. Patients who suffered PET following acute otitis media (AOM) and OME were reported by Tsuji et al [20]. They predicted that middle ear inflammation would be followed by ET mucosal inflammation and a propensity for stenosis at AOM or OME. Reduced inflammation of the ET mucosa occurs along with otitis media healing. They hypothesize that depending on how the inflammation subsides, ET mucosal fibrosis takes place, resulting in a pathologically PET. Additionally, according to Ward et al., 25.8% of PET patients had previously experienced recurrent otitis media [7]. They thought that clinicians needed to be aware that a patient with long-term dilatory dysfunction could transition to PET and acquire "burned-out" localized mucosal atrophy. These previous reports suggested that, although histological studies are needed, it is feasible that PET may be caused by inflammation of the ET mucosa due to OMAAV. There is abundant room for further progress in determining the precise mechanism of PET due to OMAAV.

## Conclusions

In this article, we described two cases of PET in patients experiencing otitis media with OMAAV.

Body weight loss due to ANCA-associated vasculitis causes PET. Another potential cause is inflammation of the ET mucosa due to ANCA-associated vasculitis, which may lead to ET patency due to atrophy of the ET mucosa. ANCA-associated vasculitis causes pulmonary, renal, neurologic, cutaneous, and gastrointestinal lesions as well as facial nerve palsy and hypertrophic pachymeningitis. Further study is needed to determine what proportion of PET cases are present in OMAAV.
